# Disentangling Tinnitus Distress and Tinnitus Presence by Means of EEG Power Analysis

**DOI:** 10.1155/2014/468546

**Published:** 2014-09-03

**Authors:** Martin Meyer, Matthias S. Luethi, Patrick Neff, Nicolas Langer, Stefan Büchi

**Affiliations:** ^1^Neuroplasticity and Learning in the Healthy Aging Brain (HAB LAB), Institute of Psychology, University of Zurich, Andreasstrasse 15/2, 8050 Zurich, Switzerland; ^2^International Normal Aging and Plasticity Imaging Center, University of Zurich, Zurich, Switzerland; ^3^University Research Priority Program “Dynamics of Healthy Aging”, University of Zurich, Zurich, Switzerland; ^4^Cognitive Psychology Unit (CPU), University of Klagenfurt, Klagenfurt am Wörthersee, Austria; ^5^Biological Psychology, Institute of Psychology, University of Zurich, Zurich, Switzerland; ^6^Institute for Computer Music and Sound Technology (ICST), University of the Arts (ZHdK), Zurich, Switzerland; ^7^Laboratories of Cognitive Neuroscience, Division of Developmental Medicine, Department of Medicine, Children's Hospital Boston, Boston, MA, USA; ^8^Harvard Medical School, Boston, MA, USA; ^9^City College New York, New York, NY, USA; ^10^Child Mind Institute, New York, NY, USA; ^11^Clinic for Psychotherapy and Psychosomatics “Hohenegg”, Meilen, Switzerland

## Abstract

The present study investigated 24 individuals suffering from chronic tinnitus (TI) and 24 nonaffected controls (CO). We recorded resting-state EEG and collected psychometric data to obtain information about how chronic tinnitus experience affects the cognitive and emotional state of TI. The study was meant to disentangle TI with high distress from those who suffer less from persistent tinnitus based on both neurophysiological and behavioral data. A principal component analysis of psychometric data uncovers two distinct independent dimensions characterizing the individual tinnitus experience. These independent states are distress and presence, the latter is described as the perceived intensity of sound experience that increases with tinnitus duration devoid of any considerable emotional burden. Neuroplastic changes correlate with the two independent components. TI with high distress display increased EEG activity in the oscillatory range around 25 Hz (upper *β*-band) that agglomerates over frontal recording sites. TI with high presence show enhanced EEG signal strength in the *δ*-, *α*-, and lower *γ*-bands (30–40 Hz) over bilateral temporal and left perisylvian electrodes. Based on these differential patterns we suggest that the two dimensions, namely, distress and presence, should be considered as independent dimensions of chronic subjective tinnitus.

## 1. Introduction

Tinnitus is an auditory phantom percept of chronic high-pitched sound, noise, or ringing, typically in the frequency range of 6–8 kHz, without any objective external sound source [1]. For this reason, reliable, objective measures of tinnitus are difficult to obtain and require sophisticated acoustic and psychometric techniques. Despite patients' occasional descriptions of immensely loud and tantalizing sounds, it has been shown that tinnitus occurs at intensities only 5–10 dB above hearing threshold [2]. In Western industrial countries with a steadily aging population, the number of individuals who suffer from tinnitus is immense. According to Cederroth and colleagues [3] approximately 50 million people in the US and 70 million individuals in the European Union, that is, approximately 10% of the population, are affected. Meanwhile, it is widely accepted that tinnitus must not be considered as a sole dysfunction of the inner ear even though tinnitus is normally preceded and accompanied by transient or permanent hearing loss [2, 4]. It has rather been agreed that tinnitus emanates from a perplexing network that includes the ear and the auditory pathway but primarily resides in the human brain [5–9]. Tinnitus is highly subjective in nature and for this reason it is not considered as a physical disease but a heterogeneous diffuse phenomenon that lacks a clearly defined neurological pathogenesis [10]. Thus, it comes as no surprise that several, partly conflicting, neurobiological models exist that sketch the complex interplay between multiple cortical and subcortical human brain areas which may mediate the subjective experience of chronic tinnitus [6, 10–14]. These models agree in that they describe tinnitus as the unwanted result of an imbalance between inhibition and excitation of thalamocortical circuits [15]. According to this view, peripheral hearing loss caused by damage to hair cells in the inner ear results in deficient auditory information transfer to the auditory brain. The loss of sensory input instantiates low frequent self-oscillations of thalamic cells which activate the auditory cortex. This aberrant pattern of activity becomes fortified in thalamocortical feedback loops [12]. In analogy to the aching “phantom limb” sensation, tinnitus can also be considered as a phantom pain phenomenon that results from neuroplastic alterations during remapping of the auditory cortex [16]. To this end, tinnitus should be viewed as an unwanted perceptual state and function of incremental maladaptive learning. In the absence of externally generated inflowing information the phantom sound sensation is gradually but steadily reinforced internally. Top-down processes of attentional allocation become more and more dominant because concerned individuals are increasingly irritated and become aware of this disturbing noise in their heads. In absence of any reasonable and appropriate coping strategies these persons consider the permanent sound as extremely detrimental. Consequently, the neural thalamocortical circuit that maintains the phantom sound connects with attentional circuits. This neural loop is accelerated by aversive emotional attributions, is continuously updated, and eventually becomes established. Thus, chronic subjective tinnitus could be considered as a learned disorder that results from maladaption of several overlapping brain systems that bind together in a “vicious circle” [6].

To date there is no medical, neurological, or neuropsychological therapy that has been proved as universal treatment to cure tinnitus [4]. This lack of a standard treatment can be taken as an obvious evidence that subjective tinnitus is an exceptionally dynamic and complex phenomenon that emerges from a cascade of neuroplastic processes. In vivo morphometry studies indirectly also confirmed this notion in that they draw a complex picture of the structural neuroarchitecture of tinnitus. According to these studies a loose ensemble of cortical and subcortical limbic brain regions in TI have increased or decreased in volume, thickness, or surface [17–23]. One recent study applied an advanced approach in that the authors correlated neuroanatomical traits with tinnitus-related distress within a TI large sample [24]. According to these authors an inverse relationship between cortical volume in bilateral auditory areas and distress can be observed. However, due to the constant emission of detrimental scanner noise and other uncomfortable aspects of scanning environment many TI are reluctant towards participation in MRI studies so that magnetic resonance imaging cannot be considered as a suitable technique to explore the functional signature of tinnitus.

Alternatively, spectral power and connectivity analyses of resting-state EEG have been turned out as advantageous tools because recent research has demonstrated that EEG parameters obtained from TI generally deviate from EEG patterns of people without tinnitus symptoms [25]. Enhanced EEG activities in *δ*-, *θ*-, and *β*-bands have been described as indicative of a chronic dysrhythmia of thalamocortical circuits following auditory deafferentation. Along the same vein, chronic tinnitus has been associated with increased *γ*-band activity in the contralateral auditory cortex [26, 27]. However, tinnitus appears not only to affect neural circuits in the auditory cortex but also ensembles residing in the associative/paralimbic system, anterior cingulate, insula, prefrontal cortex, posterior cingulate, and (pre)cuneus [9]. To date several studies have been published that sought to identify deviant profiles in neurophysiological or neuromagnetic recordings that help better describe the interplay between different resting state networks that partly overlap and form larger oscillatory networks, thereby amalgamating brain circuits and form novel—partly maladaptive—associations [6, 11]. With respect to the *α*-band the situation is less clear. In TI both decrease and increase of *α*-activity have been observed [11]. While some authors surmise that a decay of *α*-oscillations mandatorily precedes an increase of *γ*- and *θ*-oscillations as a function of dysrhythmia, other scholars conjecture that an observed increase of *α*-activity is a proactive mechanism of the brain to suppress other tinnitus-related EEG frequencies [11].

Interestingly, a fraction of, but not all, individuals suffering from subjective tinnitus also show symptoms of psychiatric disorder and moderate symptoms of depression or anxiety or indicate considerable emotional distress. Zöger and colleagues [28] report a high prevalence of psychiatric, clinically pertinent diagnoses in a sample of TI. Depressive (62%) and anxiety (45%) disorders were noted in the investigated TI population underlining the paramount importance to carefully identify these affective disturbances in individuals suffering from subjective chronic tinnitus. More recent approaches using power spectrum analysis aim at correlating clinically pertinent psychometric measurements with specific increases or decreases of frequency-band-specific oscillatory modulations [10, 29–34]. Three issues are primarily discussed as reliable predictor variable, namely distress, duration, and loudness. Duration is understood as the amount of time that has passed from onset of the tinnitus sensation until the measurement. This variable can be assessed quite easily. Loudness (or intensity) is more difficult to measure. It can be either assessed by means of a visual analog device [34] or by means of an acoustic tinnitus simulation. In the latter case TI are able to adjust the subjective loudness of their individual tinnitus sensation. By means of standard questionnaires [35], distress has been identified as the most pertinent predictor [11]. Recent studies describe distress as a serious and grave dimension [34]. Apparently, distress can be delineated as an emotional state that is frequently, but not necessarily, coupled with tinnitus because distress may be present in TI, but its strength may be completely independent from duration, loudness, or depression [11, 29–31]. 

*The Present Study*. The aim of the current study is to further explore the complex interplay between the multitude of variables that contribute to the heterogeneity of tinnitus. We investigated individuals suffering from tinnitus and nonaffected persons. However, our main interest comprises the heterogeneity of psychological and neural patterns of tinnitus. To account for this heterogeneity we did not take the standard approach (comparing tinnitus patients and control subjects). We rather thoroughly elucidate the differential psychopathological and neurophysiological individual profiles within a population of TI. Our psychometric toolbox includes standard questionnaires on tinnitus experience, depression, and other biographical details (see Section 2.3). Individual hearing thresholds will be determined to control the effect that hearing loss may have on distinct components in the tinnitus network. Furthermore we will test the application of a nonverbal self-evaluation for pictorial representation of illness and self measure (PRISM) [36, 37] as an alternative instrument to determine the relevance of tinnitus in the life of concerned individuals.

Akin to Vanneste and coworkers [34] a principal component analysis (PCA) is used to identify the independent dimensions underlying our comprehensive psychometric data. This procedure is advantageous in that it is devoid of any a priori constraints about latent relationships between behavioral variables.

Additionally, resting-state EEG was recorded to investigate whether the identified behavior and self-evaluation-based components may predict distinct neural signatures in the EEG power spectrum. Increase in *γ*-power, presumably generated in the auditory regions, has been discussed as manifestation of activity in the core tinnitus network [11, 27]. There is uncertainty regarding the role of the *α*-band originating from auditory fields which has been observed to increase and decrease in TI. According to Vanneste and collaborators [34], the auditory component may explain only 4-5% of total signal variance while other components, that is, contributions of extraauditory circuits, may account for the remaining variance. Tinnitus-related emotional distress has been associated with EEG modulation preponderance at different oscillations, namely, *α*-band [30, 32, 34], *β*-band [29, 32, 34], and *γ*-band [34]. With respect to this incongruous scheme and due to the data-driven PCA-based approach we only devise careful predictions. First, we hypothesize that the PCA will identify independent dimensions that underlie the psychometric data within the TI population. The identification of these traits will help better distinguish between differential tinnitus profiles. Secondly, we predict a significant relationship between the independent components and corresponding distinct neural signatures that can be evinced by EEG power spectrum and topographical maps of EEG signal scalp distribution. Finally, we expect a correlation between an established verbal and a not yet established nonverbal self-evaluation tool (PRISM). The investigation of the latter in the context of diagnosis of tinnitus is novel and may have interesting implications as the application of PRISM, the nonverbal device, takes only a fraction of time relative to the standard verbal tinnitus questionnaire.

## 2. Methods

### 2.1. Participants

We recruited a sample of 24 TI (age *M* = 39.75, SD = 12.11, and range 20–62) and 24 control subjects without tinnitus (CO; (age *M* = 37.04, SD = 9.97, and range 20–62)). All participants were comprehensively informed about the background and the aim of the study. They all gave written informed consent.  Table 1 shows the demographics and clinical details of TI. As apparent from this table the included TI suffer from tinnitus of heterogeneous origins. Right- and left-handed individuals were accepted for the study, as there were no indications for a relation between tinnitus laterality and handedness. Tinnitus severity, as assessed by the Tinnitus Questionnaire (TQ), varied between 4 and 65 (TQ total score range 0–84, with higher scores indicating higher grades of distress, *M* = 26.75, and SD = 16.43). No participant was reported to suffer from any neurological disorder. The control group was matched to the tinnitus group with regard to age, years of education, sex, handedness, musical background, and time of day during the EEG recording. The years of education in the tinnitus group (*M* = 17.83, SD = 3.87) did not differ from the control group (*M* = 17.88, SD = 4.08  *t*(46) = −.036, and *P* = .971). All volunteering participants gave written informed consent. The study is in accordance with the ethical principles that have their origin in the Declaration of Helsinki, adopted and revised by the World Medical Association.

### 2.2. Design

The study design combined between-subjects and within-subject approaches. The independent variable for the between-subject comparisons consisted of the two levels TI and CO. As dependent variables questionnaire scores and EEG measurements in the conditions resting state (“eyes open” (EO) and “eyes closed” (EC)) were used. Since the comparison between TI and CO without consideration of specific differences in individual psychopathology did not open up compelling insights, the main focus of the analysis was on comparisons and differences in individual psychopathology within TI. Hence, correlational analyses between perceptual characteristics of tinnitus and EEG data were conducted.

### 2.3. Questionnaires

A range of questionnaires was applied to assess multiple psychological variables, namely, depression and emotional burden induced by tinnitus in all TI.

To assess tinnitus-related information, a German adaptation of the “Tinnitus Questionnaire” (TQ) [38] and a questionnaire of our own design were used. TQ is the most extensively used device to assess tinnitus-related distress [32, 39–42]. It comprises 52 statements, which are judged on a three-point Likert scale (“true,” “partially true,” and “not true”). Besides a total score for tinnitus distress and severity, six subscores (“Psychic Distress,” “Intrusiveness,” “Auditory Perceptual Difficulties,” “Sleep Disturbances,” and “Somatic Complaints”) are derived. The subscore “Psychic Distress” is further subdivided into “Emotional Distress” and “Cognitive Distress.” Our own questionnaire collected information about the tinnitus such as origin, duration, perceived side effects, or previous treatment options.

To determine hearing thresholds in all participants, we used the Home Audiometer software [43] in a sound-proofed room.

### 2.4. Distress Rating Tools

#### 2.4.1. Beck Depression Inventory

Signs of depressions were measured by means of “Beck Depression Inventory” (BDI) [44]. The BDI contains 21 items which assess various symptoms of depression. The sum score over all items imply the degree of depressive mood or depression.

#### 2.4.2. The PRISM Task

**Figure 1:**
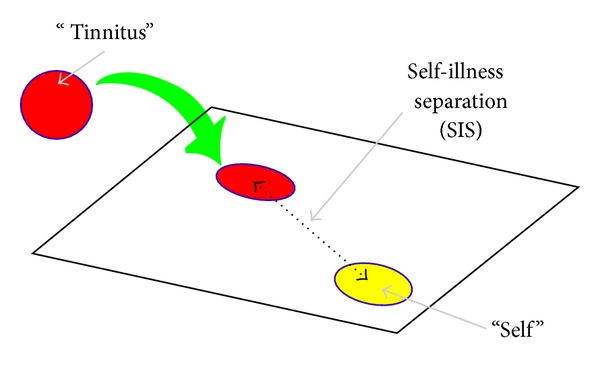
Self-evaluation of individual tinnitus-related distress by means of PRISM. TI imagined a metal board representing her life and a small yellow circle on the board representing her self. The task was to place a small magnetic red disk on the board to indicate the current salience and distress of tinnitus in the patient's life. Afterwards, the distance between the self and the tinnitus disk was measured as a quantitative measure of the burden of individual distress.

PRISM has been validated to measure burden of suffering in a variety of chronic diseases [37, 45–47] and was applied in this study as described elsewhere [36, 48]. Briefly, the patient is shown a white A4-sized metal board with a fixed yellow disk (representing the patient's self) at the bottom right-hand corner and asked to imagine that the board represents her life as it is at present (see Figure 1). The patient is then handed a red disk, asked to imagine that this represents her illness, and then asked one question: “Where on the board (representing your life) would you place the disk (representing tinnitus) at the moment?” The purpose of this task is to reflect the importance of the illness in her life. The main quantitative measure derived from PRISM is the self-illness separation (SIS), namely, the distance in millimeters between the centers of the illness disk and the self disk.

#### 2.4.3. Visual Analog Scale

We applied “visual analog scales” (VAS) as a tool to measure the current tinnitus sensation. Participants rated the instantaneous strength of their tinnitus sensation on a paper sheet several times during the recording session. We opted for the term “strength of tinnitus sensation” in an attempt to capture the full qualitative spectrum of tinnitus perception, including loudness, disturbance, and intensity.

### 2.5. EEG Recordings

The recordings were made utilizing a dense array EEG system with 129 channels and were saved electronically with Net Station, both developed by Electrical Geodesics, Inc. [49]. The sampling rate was set to 500 Hz and impedances were kept below 40 kΩ. The CZ electrode was used as reference for online recording.

#### 2.5.1. Procedure

Presentation software [50] was used to control the measurement procedure. Participants were informed about the course of events before the recording session was started. They were instructed to assume a comfortable position in the chair and to remain calm for the recording. EEG parameters were continuously monitored and checked for abnormalities during the recording sessions. A resting EEG was obtained during six minutes. It consisted of alternately 20 seconds of EO and 40 seconds of EC. Subjects were instructed via a prerecorded voice to open or close their eyes, respectively. During the EO periods, a cross mark was shown on the computer screen and participants were instructed to fixate it. TI were subsequently asked to rate the strength of the tinnitus sensation for EC and EO separately on a VAS (ranging from “not strong at all” to “very strong”). The EO periods are primarily meant to maintain a constant level of vigilance.

### 2.6. Data Analysis

#### 2.6.1. Behavioral Data and Questionnaires

For TQ, we put special emphasis on the total score and the subscore of “Emotional Distress” because these two measures reliably predict tinnitus-related distress. In an exploratory manner, several variables of tinnitus distress were correlated with various scores of tinnitus strength, tinnitus duration, tinnitus pitch, tinnitus loudness estimations, and further behavioral variables addressed by the questionnaires. According to the study by Schlee and collaborators [39] who showed that individual tinnitus duration significantly contributed to tinnitus-related brain activity, we identified* tinnitus presence* as an important component for further analysis. To this end, tinnitus duration was transformed to the total amount of months to gain a parametric scale. Tinnitus pitch estimations (as obtained by means of sine wave generator of the MAX software suite [51]) were included to test whether it shares commonalities with the other subjective tinnitus variables.

#### 2.6.2. Preprocessing of EEG Data

The raw data files from Net Station were transformed into EDF file format in order to preprocess them with BrainVision Analyzer [52]. Butterworth zero phase filters were applied: low cutoff was set at 0.5 Hz and high cutoff at 100 Hz. A notch filter was implemented at 50 Hz to reduce effects of the electric circuit on the EEG signal. For the PCA, the entire data set of each participant was used. Components containing eye movements or heart beats were identified and removed after a visual check of their impact on the EEG signal. Topographical interpolation was performed in order to recalculate rejected channels based on the signal mean of the four nearest electrodes. Next, the signal was re-referenced to the average amplitude of all electrodes at each sampling point (average reference). Data was segmented into 2 s epochs. After identification of remaining artifacts (e.g., muscle artifacts) based on visual inspection and supporting algorithms, respective segments were excluded from further processing. For EC this procedure resulted in a range of 72 s to 238 s of analyzable EEG data (*M* = 185.54, SD = 36.01).

#### 2.6.3. Power Spectral Analysis

The number of electrodes was reduced to 109 channels by omitting the outermost ring of electrodes, as they usually show high amounts of noise. A power spectral analysis was applied using Fast Fourier transformation (FFT) with a Hanning window of 10% segment length. The spectrum from 0.5 Hz to 45 Hz was used with a resolution of 0.5 Hz. The power spectrum was averaged over all available 2 s segments for each subject. Next, segments were averaged over all available segments for each subject and electrodes separately. Data was then exported to MATLAB [53] for statistical analysis with custom scripts.

In analogy to the behavioral data, statistical comparisons between groups, conditions, and regression analyses were conducted. Student's *t*-tests were used to compare TI to CO during resting state. One-tailed tests were applied as we expected higher power values in the TI. Tests were done for each electrode and frequency bin separately. Behavioral data and EEG data were correlated by means of Pearson product-moment correlations within the TI population and cross-validated with nonparametric tests where indicated. Variables were controlled by means of partial correlations where appropriate. We generated topographical maps on the basis of the outcome of the statistical analysis. The topographical maps visualize the mean correlation between one of the two components and spatial distribution of EEG signal distribution across the scalp.

## 3. Results

### 3.1. Behavioral Data

For all questionnaire variables, psychometric data between TI and CO were compared. For the BDI, TI (*M* = 11.63, SD = 10.21) exhibited a higher extent of depression than the CO (*M* = 4.04, SD = 5.64, *t*(36.16) = 3.167, and *P* = .003). Of additional interest, BDI scores varied more strongly in TI relative to CO (*F*(45) = 5.045, *P* = .030). A closer look revealed that 12 TI (out of 24) and 21 CO (out of 23, missing data from one control subject) showed normal scores.

To analyze the audiometric assessment, an average hearing threshold was built across all measured hearing thresholds (125, 250, 500, 1000, 2000, 4000, 6000, and 8000 Hz) for each ear separately. We found the average hearing threshold to be significantly higher in TI (*M* = 18.98, SD = 7.47) than in CO (*M* = 13.07, SD = 3.63, *t*(33.29) = 3.489, and *P* = .001).

Correlations were computed between all subjective measures of tinnitus characteristics and all questionnaire variables. A summary of the relevant data can be seen in Table 2. Interestingly, the PRISM tool has turned out to be highly indicative of tinnitus-related burden, as it correlates highly with TQ total score (*r* = .763,  *P* < .001) and with TQ subscale “Emotional Distress” (*r* = .728,  *P* < .001), with VAS (*r* = .498,  *P* < .013) as well as with BDI (*r* = .423,  *P* < .039). Tinnitus loudness (not depicted) did not correlate with other tinnitus-related variables. However, tinnitus pitch correlated positively with all measures related to tinnitus distress and tinnitus VAS scores on a moderate level. The highest correlation was observed between pitch and “Emotional Distress” (*r* = .696,  *P* < .001).

#### 3.1.1. Principal Component Analysis

As apparent from Table 2, the measures for tinnitus-related distress (TQ, PRISM, and tinnitus disturbance) showed intercorrelations on a moderate to high level. Thus it can be assumed that they are valid traits of tinnitus distress. Furthermore this pattern suggests that a single dimension may account for the majority of variation in the variables. Tinnitus duration correlates negatively with all measures of tinnitus distress but correlates positively with subjective strength of the tinnitus sensation as measured by the VAS. We thus concluded that other dimensions besides tinnitus distress contribute to the present strength of the tinnitus sensation. In order to extract converging information of the different psychometric measurements of tinnitus, we explored the available data with a PCA. PRISM, VAS, tinnitus duration, and TQ total score were included in the PCA. For the TQ, we decided to focus on the total score and not on “Emotional Distress” because items from other subscores also contained information relevant for evaluating the burden of suffering (e.g., item 10, which belongs to the subscale “Intrusiveness”: “The ear sounds are really unpleasing.”). The Kaiser-Meyer-Olkin measure (KMO) verified the sampling adequacy for the analysis; KMO = .55 [54]. Bartlett's test of sphericity, (*P* < .001), indicated that correlations between measures were sufficiently large for PCA. The extracted components were rotated with Varimax rotation with Kaiser normalization. An initial analysis revealed two components with eigenvalues >1, and the scree plot showed an inflexion which also justified to retain two components. In total the two components accounted for 85% of the total variance, which is satisfactory.  Table 3 summarizes the results from the PCA.

The two applied measures of tinnitus-related distress (PRISM, TQ total score) and tinnitus strength as measured by VAS loaded strongly positively on the first component. We considered the first component to capture tinnitus-related distress, that is, the amount or burden of subjective suffering caused by the tinnitus. Tinnitus strength as measured by VAS loaded also strongly positively on the second component, together with tinnitus duration. We interpreted the second component as tinnitus presence, a perceptive aspect of tinnitus capturing the conscious awareness of the noise which increases with tinnitus duration but is unrelated to tinnitus distress. Of note, tinnitus strength as quantified by VAS is the only variable that loaded on both components, distress and presence. Based on the pattern of results we reasoned that there is a part of the tinnitus experience that is emotionally neutral, as expressed in the second component. Akin to our approach Schlee and colleagues [39] observed brain responses in TI that correlated with tinnitus duration, but not with tinnitus distress.

As apparent from Table 4 the two components do not correlate with the hearing threshold. This finding allows an interpretation of the data independent of the individual extent of hearing loss. While distress does not correlate with age we observed a weak correlation between age and presence for obvious reasons (*r* = .380, *P* = .067). BDI scores show a significant positive correlation with distress but not with presence. These findings support our interpretation that the first component delineates tinnitus-related distress whereas the second component depicts an emotionally neutral aspect of tinnitus. In conclusion, we identified two independent components that comprehensively explain the experience of subjective tinnitus in our sample of TI.

#### 3.1.2. EEG Data

We analyzed EEG data for both EO and EC separately. The results between these two conditions did not differ considerably. Hence, we only report analyses for EC condition because it contains fewer artifacts from eye and head movements.

**Figure 2:**
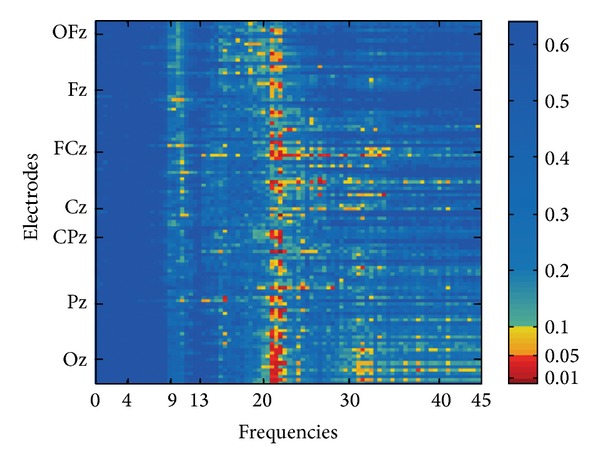
Comparison of TI versus CO normalized EEG spectral power adjacency matrix of the group comparison (TI versus CO): significance levels (*P* values) of the correlations are color-coded (one-sided). On the *y*-axis electrodes are aligned from rostral (top) to caudal (bottom), irrespective of laterality. Positions of electrodes on the central line are marked.

**Figure 3:**
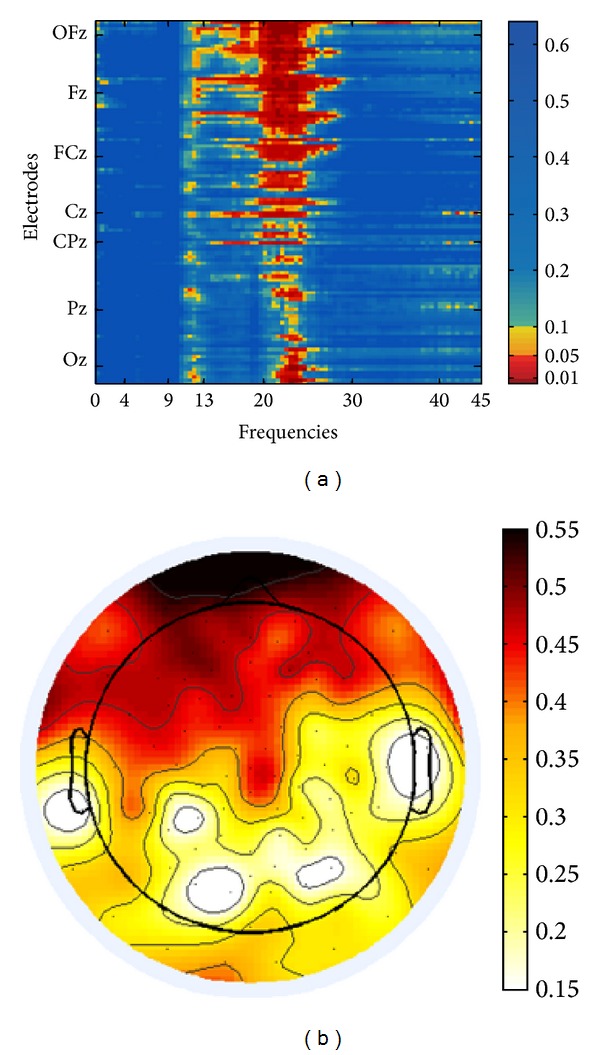
(a) Nonnormalized EEG spectral power adjacency matrix of Pearson product-moment correlations with tinnitus distress. Significance levels (*P* values) of the correlations are color-coded (one-sided, uncorrected for multiple comparisons). On the *y*-axis electrodes are aligned from rostral (top) to caudal (bottom), irrespective of laterality. Positions of electrodes on the central line are marked. (b) Topographic plot of EEG power correlations in the upper *β*-band (20–25 Hz) with* tinnitus distress* and strength of correlations (Pearson's *r*) are color-coded.

**Figure 4:**
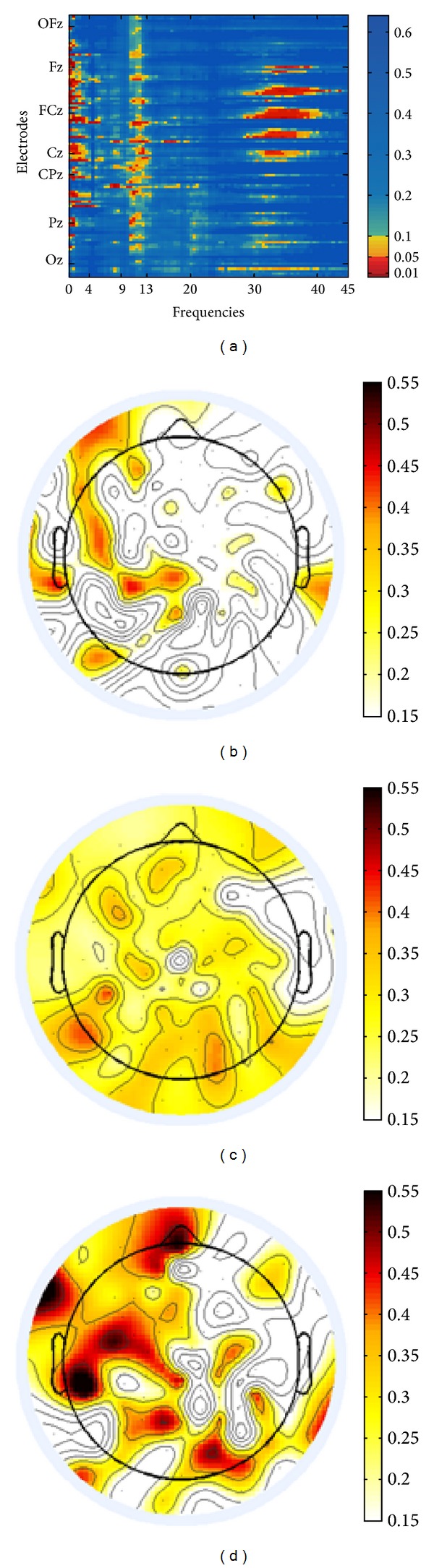
(a) Nonnormalized EEG spectral power adjacency matrix of Pearson product-moment correlations with tinnitus presence. Significance levels (*P* values) of the correlations are color-coded (one-sided, uncorrected for multiple comparisons). Electrodes on the *y*-axis are aligned from rostral (top) to caudal (bottom), irrespective of laterality. Positions of electrodes on the central line are marked. (b)–(d) Topographic plots of EEG power correlations in the *δ*-band (0.5–4 Hz) (b), in the *α*-band (9–12 Hz) (c), and in the *γ*-band (30–40 Hz) (d) with* tinnitus presence* and strength of correlations (Pearson's *r*) are color-coded.

Figures 2–4 present the topographic maps and results of FFT-based power analyses. Please note that Figure 2 depicts normalized EEG power between the TI and CO groups, while Figures 3 and 4 show nonnormalized EEG pattern with the TI group. For the comparison between TI and CO segments were normalized by dividing each frequency bin by the total area under the curve of the according spectrum, thereby ascertaining that the total power of each spectrum was one unit. As apparent from Figure 2 TI showed weakly increased EEG power in the upper *β*-band between 20 and 22 Hz compared with CO. However, as outlined above, we did not expect substantial differences between TI and CO as the first group cannot be considered homogeneous due to the independent components—distress and presence—we identified. Thus, we did not further explore the difference between TI and CO but analyzed the two component scores obtained from the PCA. For this procedure, we used the components distress and presence to correlate behavioral data and the EEG signal.

The EEG data partly confirmed this data-based differentiation. Negative correlations with tinnitus distress and presence, respectively, were not observed. Both Figures 3 and 4 visualize positive correlations between components and EEG signal. For the analyses of FFT-based power of TI we refrained from normalization of EEG data because we consider normalization only necessary when datasets from different groups are compared. Furthermore the results obtained from both normalized and nonnormalized data did not differ qualitatively. With respect to tinnitus-related distress we observed a strong correlation between distress and the EEG signal in the oscillatory range between 20 and 25 Hz (upper *β*-band) (cf.  Figure 3). As apparent from the topographical map the maximum of activity agglomerates over frontal electrodes. A differential pattern of responses was observed for the second component. We performed partial correlations between presence and the EEG signal to control for age effects as this component weakly correlated with age (*r* = .380, *P* = .067). Presence corresponded to enhanced EEG signal predominantly in the *δ*-band (0.5–4 Hz), *α*-band (9–13 Hz), and lower *γ*-band (30–40 Hz) (cf.  Figure 4). Unlike the first component, presence corresponded to a bilateral, but left dominant, maximum of activity over temporal auditory and adjacent left extraauditory recording sites for all frequency bands in general, but with a salient maximum for *γ*-activity.

Hence, the topographical maps and the EEG power analysis clearly confirm the results of the behavioral data. In the TI population differential subgroups can be identified who show differential psychometric and neurophysiological profiles.

## 4. Discussion

Even though 5–10% of the population in Western countries suffer from chronic tinnitus, it is bewildering to realize that only little is known about the neuroplastic changes and individual stamping of this phenomenon. This holds in particular because a significant fraction of TI (approximately 20%) [30] develop serious symptoms of distress that gravely affect their quality of life. The major reason for the immense lack of knowledge is the vast heterogeneity of tinnitus generators, the individual severity of primary and secondary, comorbid symptoms, the differences in individual coping strategies, and attribution of the life-illness relationship. Tinnitus is a phenomenon that is indicated by one sole major symptom, namely, a constant ringing in the ears or in the head but may manifest itself in multiple different forms and conditions. A recent integrative framework of auditory phantom perception proposes a compelling view that describes the interplay of several separable subnetworks in the human brain that are involved in tinnitus experiences [11]. According to this model tinnitus can be understood as a “unified coherent percept” that is modulated by a complex compound consisting of various psychological and neural traits. Moreover, correlating psychometric and clinical traits with EEG signal modulations offer a powerful option to systematically research the differential tinnitus profiles.

According to this framework we performed a study which collected psychometric, biographical, and neurophysiological resting state data in order to elucidate underlying mechanisms and opaque relationships between behavioral traits and EEG signal modulations.

The TI and CO samples are well matched in sex, age, handedness, and other biographical variables. Within the two samples we had an even distribution of age with approximately 25% of participants in each out of four lifetime decades. Based on this distribution age-biased effects (irrespective of hearing loss) in the TI population can be excluded. However, it should be mentioned that hearing loss was more prominent in the TI compared to the CO group. As apparent from Table 1 the TI indicate various effective or apparent elicitors that may have caused chronic “auditory pain.” It thus comes as no surprise that we eventually identified two independent factors that characterize tinnitus.

The correlations between various tinnitus-related measures yield a plausible pattern (cf. Table 2). Generally, we noted a dense relationship between TQ total score, a comprehensive measurement of tinnitus-related annoyance, and BDI scores that indicate symptoms of depression. The same holds for other applied self-evaluation tools of subjective distress, namely, VAS and PRISM. A study by Joos and colleagues [30] provided evidence for the view that depression and distress could be disentangled in TI and are likely to recruit distinct neural circuits. However, they report a significant positive correlation between BDI and mini-TQ (*P* < .05) which is generally indicative of an existing relationship between these two emotional states. Maybe the fact that they used a reduced version of the TQ may account for the weaker correlation they observed. Based on our results we cannot make any statements about potential distinct neural circuits that mediate depressive symptoms in TI. Notably, Joos and colleagues observed correlations between activity in the *β*-band and both BDI and mini-TQ scores. This observation is similar to our finding of increased EEG activity in the *β*-band in TI who suffer from emotional distress. Thus, we suggest that tinnitus can be accompanied by both a transient state of distress and annoyance and a more constant depressive mood. Likely, the availability of appropriate coping mechanisms is supposed to modulate this relationship [55].

PRISM has been used successfully in several different clinical settings to gain a better understanding about the self-assessed relationship between a patient and their illness [37, 45–47]. The high correlations between PRISM and other tinnitus-related measures, namely, TQ (*r* = .763,  *P* < .001), VAS (*r* = .498,  *P* < .013), and BDI (*r* = .423,  *P* < .039), are in line with our predictions. PRISM scores showed the highest loading on the first PCA component distress, providing further evidence for the pertinent role of PRISM in measuring tinnitus distress (cf.  Table 3, (.907)). Hence, we suggest that PRISM may be a quick, easy, and effective alternative to the application of the verbal TQ. Evidently, PRISM nonverbally measures tinnitus-related distress by means of solely one perspicuous question and achieves a validity that is similar to the TQ but takes considerably less time and is easy to perform.

Another interesting finding pertains to the relationship between tinnitus pitch, self-assessed by a standard sine wave generator to measure the approximate individual pitch height of the chronic noise (cf. Table 2). Tinnitus pitch correlates significantly with TQ total score (*r* = .589,  *P* < .004), TQ “Emotional Distress” (*r* = .696,  *P* < .001), PRISM (*r* = .576,  *P* < .005), and BDI (*r* = .475,  *P* < .026). In other words this finding suggests that the higher the subjective tinnitus the higher is the amount of distress. In our view this relationship has not yet been observed before and it is by all means worth reporting because it may imply that the determination of tinnitus pitch might reflect the interplay between subjective distress and objective features of the percept. 

*The First Component: Tinnitus-Related Distress.* By means of comprehensive psychometric and neurophysiological data we identified two independent components that are supposed to characterize different TI profiles. The first component, “distress” can be straightforwardly interpreted because of the high loadings of PRISM, TQ-evaluated annoyance, and VAS. Across several psychometric measurements this component explains a high amount (55%) of the data. Interestingly, distress does not correlate with hearing loss and thus is probably not mechanically linked to the deafferentiated and dysfunctional auditory system. Previous studies that also applied hypothesis-blind approaches have identified a variable termed “distress” or “annoyance” [29, 34]. Thus, it is plausible to reason that tinnitus is frequently but not necessarily all the time related to transient emotional distress.

According to our results there is a relationship between the strength of distress and neural modulations in the upper *β*-band (20–25 Hz). While the tinnitus percept is frequently reported in association with *γ*-band increase [27, 31], studies that particularly elucidated the neural underpinnings of tinnitus-related distress noted changes across the entire *β*-band [29, 30]. In comparison to CO without tinnitus percept Vanneste and colleagues noted increased *α*- and *β*-oscillations originating from the dorsal anterior cingulate cortex in TI with distress [32]. Akin to our finding Joos and coauthors [30] observed in TI with unilateral tinnitus the occurrence of *β*-waves, predominantly in frontal areas that showed a strong positive correlation with distress. However, our finding of *β*-band activity is not perfectly comparable to the aforementioned studies as the present study does not provide results obtained from source estimation. This constraint notwithstanding the topographical map (cf.  Figure 3(b)) shows the maximum of the distributed activity over frontal recordings sites which strongly speaks in favor of involvement of frontally situated top-down mechanisms that are recorded primarily when individuals pay attention to internal or external (auditory) stimuli [11, 30]. Presumably, dominant frontal signal power may be indicative of top-down driven evaluation processes that are closely related to the distress condition. However, based on our present data, it cannot be proposed whether this signal increase reflects successful coping with the tinnitus percept (competence) or whether it corresponds to a strenuous but unavailing attempt to get along with the disturbing sensation of chronic noise (incompetence). Actually we consider the latter interpretation more reasonable in the context of the existing literature. In their position paper De Ridder and coauthors [11] also suggest that preponderant *β*-oscillations can be attributed to dysfunctional noise canceling mechanisms. In our view this explanation can be brought in line with both the first and the latter interpretations. However, it should be mentioned that the distress circuit obviously at work in TI is not specifically related to the tinnitus percept but is probably identical with the general distress network that is part of a large-scale brain system. This network is supposed to mediate other aching percepts, namely, chronic pain [6, 56]. 

*The Second Component: Tinnitus Presence.* The second component we unveiled and named presence captures a perceptive aspect of tinnitus sensation. This dimension of tinnitus can be clearly distinguished from distress as it does not load on the distress-sensitive measurement tools but has high loadings from tinnitus duration (.917), that is, the period of onset from tinnitus experience until the screening session (see Tables 3 and 4, Figure 4). Apparently, presence as well as long-term duration of the tinnitus percept does not necessarily result in emotional distress and annoyance. Like distress, presence only correlates weakly with hearing loss and should thus be considered as independent from hearing integrity. Hence our data indicate that the presence, that is, the conscious awareness, of tinnitus increases as a function of tinnitus duration while this relationship does not hold for distress. Probably a fraction of TI have developed appropriate coping strategies to inhibit tinnitus-related annoyance. Interestingly, the two separate dimensions of tinnitus experience also indicate that some concerned individuals habituate to the chronic noise and consider it less annoying after some time, despite increasing presence of tinnitus sensation.

The results of the power analysis in TI show a more complex neurophysiological pattern correlating with presence as compared to the distress-related EEG modulations. As apparent from Figure 4(a) we observed minor but nonetheless significant signal increase in the *δ*- (Figure 4(b)) and *α*-band (Figure 4(c)). Furthermore we noted increased oscillations in the lower *γ*-band. For this frequency band the topographical map shows a maximal distribution of local power over (predominantly) left and (less prominently) right centrolateral recording sites (Figure 4(d)) which may be indicative of neural origins residing in auditory fields. This view concurs with the model proposed by De Ridder and coworkers [11] who describe “persisting gamma activity localized in one brain area” as “pathological” (page 8). In this view *γ*-activity signals the breakdown of thalamocortical balance and reflects the binding of abnormally “distributed neural gamma activity into one coherent percept” (page 8). Thus, it should be regarded as the neural signature of abnormal synchronous oscillations, that is, the chronic tinnitus percept [12]. The occurrence of increased awareness of the tinnitus mirrored by enhanced *δ*- and *α*-activity in the EEG power spectrum can also be explained in the context of a complex network architecture. Usually *α*-waves are recorded from the auditory cortex during resting state and are indicative of a normally functioning system [11]. This statement notwithstanding increased *α*-band modulations have been observed in other former studies that investigated TI [16, 30, 57]. One possible explanation may reconcile these two apparently contradictory findings. It is conceivable that the default mode *α*-band activity serves as part of an active noise canceling system that (pro)actively eliminates detrimental noise in both TI (who are not this distressed) and nonaffected individuals. In other words, the significant *α*-band activity we revealed as a dimension of tinnitus presence can be considered a normal pattern of auditory activity that blocks any disturbing acoustic signal, amongst others' internally generated chronic noise. In TI who suffer more strongly and display more symptoms of tinnitus-related distress the noise-canceling system has been broken down due to maladaptive coping mechanisms. Increased *δ*-oscillations have also been associated with a deficient noise suppression mechanism [11] and should be considered a consequence of sensory deprivation that may result sooner in *θ*-*γ* instability and later in decoupling. 

*Limitations.* Some limitations that may narrow down the significance of the present study should be mentioned. For an appropriate comparison between TI and CO it would have been indicated to match the auditory thresholds. Since there was greater hearing loss in the TI population we cannot clearly sort out the influence that hearing loss per se may have had on the comparison of EEG signal activity between TI and CO.

We concede that the age range (20–62) in our TI sample is relatively large. Little is known about the life-long neuroplastic changes of tinnitus experience on brain structure and function, but it seems that TI with an earlier onset of tinnitus apparently display less symptoms of suffering than individuals with a later onset in life [58]. Maybe the lack of cognitive coping strategies in older adults which may be a result of normal age-related frontal atrophy may account for this finding. Even though we did not notice any significant relationship between age and other variables, namely distress, depression, or disturbance, we think that a better exploration to what extent and how chronic tinnitus experience may differently affect elderly other than young(er) TI is needed [59].

Unlike previous studies [10, 29, 30, 33, 34, 60] that have also addressed the issue of neural signatures of tinnitus-related profiles we have not performed a source estimation. Of course it would have been interesting to complement our results with estimations of the EEG source generators to better understand the perplexing compound of the various facets of tinnitus. Unfortunately, probably due to the relatively small sample size, our source estimation did not yield results that weathered the conservative statistical testing we performed.

Finally it should be mentioned that the mean TQ-based distress score in our study was relatively low (TQ = 22) compared to other studies (TQ = 40.93) [30], (TQ = 40.2) [60], and (TQ = 42) [24]. Thus, it cannot be ruled out that the potential existence of behavioral and EEG effects has even been underestimated.

### 4.1. Conclusion

The present study is novel in that it—based on principal component and neurophysiological analysis—identifies two independent psychological dimensions, namely, distress and presence, that correlate with differential symptoms of chronic tinnitus and distinct neural signatures in the EEG power spectrum. While distress can be considered as a well-established factor that affects TI to a substantial degree, tinnitus perception seems to become more stable and present as a function of tinnitus duration. Interestingly, this dimension of tinnitus sensation is independent of emotional distress. The differential neural profiles observed for the two dimensions of chronic tinnitus suggest that differential adaptive and coping mechanisms in TI do exist. Hence the study makes a significant contribution to the underinvestigated field of neuroplasticity of tinnitus because it proceeds with the most recent initiatives to better understand the differences of individual psychological and neural profiles within the TI sample. We think that this approach is more promising than investing further research in the comparison between TI and nonaffected CO because our investigation has corroborated previous observations of other groups evincing that the population of TI is extremely heterogeneous. Hence, future research should concentrate on the exploration of specific behavioral and neural profiles within TI—as it has already been introduced [55, 61, 62]—to form a basis for the development of appropriate neuropsychological treatment approaches.

## Figures and Tables

**Table 1:** Demographics and clinical details of the patient group.

Number	Sex	Age	Tinnitus				
			Localization	Duration	Quality	TQ	Cause, as reported by the patient
1	Male	28	Right ear	12 y	Noise	4	Exposure to loud music
2	Male	26	Both ears	16 y	Tone	6	Playing drums otitis media (inflammation of the middle ear)
3	Male	28	Both ears	10 y	Tone	14	
4	Female	32	Right ear	8 y	Noise	14	Noise trauma, allergies
5	Female	55	Both ears	7 y	Tone	14	?^a^
6	Male	58	In the head	38 y	Noise	15	Noise trauma
7	Female	24	Both ears	4 y 6 m	Tone	16	?^a^
8	Male	32	Right ear	15 y	Tone	16	Pressure trauma from diving noise trauma, after “power meditation”
9	Male	62	In the head	33 y	Noise	16	
10	Female	31	Both ears	10 y	Tone	20	Stress
11	Male	52	In the head	5 y	Tone	20	?^a^
12	Female	31	Both ears	4 y 3 m	Tone	21	Stress
13	Male	47	In the head	15 y	Tone	21	Exposure to loud music
14	Female	29	Both ears	2 y 9 m	Tone	23	Hearing loss exposure to loud music, stress, otitis media thyroid dysfunction, and low blood pressure
15	Female	25	Right ear	1 y 1 m	Tone	28	
16	Female	41	Both ears	7 y	Noise	28	
17	Female	33	Right ear	0 y 2 m	Noise	29	Ménière's disease
18	Male	61	In the head	13 y	Tone	31	Stress, noise trauma
19	Male	31	Both ears	16 y 4 m	Tone	39	Exposure to loud music noise trauma, high blood pressure
20	Male	50	In the head	20 y	Tone	41	
21	Female	44	Both ears	1 y 6 m	Tone	47	Stress, SSRI^b^
22	Male	46	Left ear	0 y 10 m	Noise	53	SSRI^b^
23	Male	41	In the head	20 y	Noise	61	Occurred after road accident
24	Female	47	Both ears	1 y 6 m	Tone	65	Stress, otitis media, and SSRI^b^

*Note.* TQ: total score of the Tinnitus Questionnaire. Tinnitus duration is provided in years (y) and months (m). ^a^Patient did not know what caused the tinnitus. ^b^Selective serotonin reuptake inhibitor.

**Table 2:** Correlations between various tinnitus-related measures.

		TQ total score	TQ emotional distress	PRISM	Tinnitus Pitch	Tinnitus Duration	VAS	BDI
TQ Total score	*r*	1	.971∗∗∗	.763∗∗∗	.589∗∗	−0.276	.409∗	.700∗∗∗
	*P*		<.001	<.001	.004	.192	.047	<.001

TQ Emotional Distress	*r*		1	.728∗∗∗	.696∗∗∗	−.245	.427∗	.760∗∗∗
	*P*			<.001	<.001	.248	.037	<.001

PRISM	*r*			1	.576∗∗	−.381	.498∗	.423∗
	*P*				.005	.067	.013	.039

Tinnitus Pitch	*r*				1	−.143	.380	.475∗
	*P*					.526	.081	.026

Tinnitus Duration	*r*					1	.193	−.178
	*P*						.365	.406

VAS	*r*						1	.457∗
	*P*							.025

BDI	*r*							1
	*P*							

*Note.* Pearson correlations. *N* = 24. From the TQ, only the total score and the subscore “Emotional Distress” are depicted because these scores yielded high correlations with most other measures. The BDI scale is included as the sole questionnaire measure in the table because the other measures did not correlate significantly with any tinnitus-related variables. ^∗/∗∗/∗∗∗^Significant correlation with *P* < .05/.01/.001.

**Table 3:** Rotated factor loadings.

	Component	
	1	2
PRISM	**.907**	−.247
TQ total score	**.865**	−.206
VAS (eyes closed)	**.744**	**.532**
Tinnitus duration	−.216	**.917**

Eigenvalues	2.171	1.229
% of variance	55.336	29.664

*Note.* The table lists rotated factor loadings (i.e., correlations), eigenvalues, and variance explained by each component. By convention, factor loadings above .40 appear in bold.

**Table 4:** Correlations between components and other variables.

		Tinnitus distress	Tinnitus presence	Hearing threshold	Age	BDI
Tinnitus distress (first component)	*r*	1	.000	−.024	.056	.627∗∗
	*P*		1.000	.911	.795	.001

Tinnitus presence (second component)	*r*		1	.151	.380	−.033
	*P*			.481	.067	.877

Hearing threshold	*r*			1	.594∗∗	.123
	*P*				.002	.567

Age	*r*				1	.208
	*P*					.329

BDI	*r*					1
	*P*					

*Note.* Pearson correlations. *N* = 24. ∗∗Significant correlation with *P* < .01.
